# Supplementary material for: A set of multi-entry identification keys to African frugivorous flies (Diptera, Tephritidae)
Source: Zookeys. 2014 Jul 24;(428):97–108. doi: 10.3897/zookeys.428.7366 (PMC4143993; doi:10.3897/zookeys.428.7366)
Supplement: Supplementary material 4 — Key to Capparimyia [file zookeys-428-097-s004.zip › SF4_ZooKeys_key to Capparimyia/lucid_player/help/help/manual_discard_of_entities.htm]

Manually discarding and restoring Entities


# Manually discarding and restoring Entities

During most Lucid identification sessions, Entities will be removed
from Entities Remaining to Entities Discarded as a result of the Feature
states you choose. However, you may sometimes find it useful to manually
remove Entities that you know well and are certain do not match your specimen.
Manually removing Entities that you know cannot match will make some functions
(such as Best and Prune Redundants) work more efficiently.

Entities are manually discarded by dragging them from Entities Remaining
to Entities Discarded. Manually discarded Entities are indicated by red
text. To restore manually discarded Entities, drag the manually discarded
Entities back to the Entities Remaining windows. Manually discarded Entities
will also be restored if the key is restarted.
